# Supplementary material for: Socio-medical factors associated with neurodevelopmental disorders on the Kenyan coast
Source: PLOS Glob Public Health. 2025 May 30;5(5):e0003806. doi: 10.1371/journal.pgph.0003806 (PMC12124531; doi:10.1371/journal.pgph.0003806)
Supplement: S1 Table — (DOCX) [file pgph.0003806.s001.docx]

S1 Table: Adjusted multivariable analysis of relevant parental, perinatal and neonatal factors associated with autism after adjusting for parental socioeconomic and demographic status –NDD vs TD group.

|  | Kilifi Autism Study | | | NeuroDev Study | | |
| --- | --- | --- | --- | --- | --- | --- |
| Risk factor variables | **Odds ratio [95% CI]** | **p-value** | **Risk Ratio [95%CI]** | **Odds ratio [95% CI]** | **p-value** | **Risk Ratio [95%CI]** |
| Child Male sex | Non-significant in univariable analysis | | | 1.62 [0.89, 2.94] | 0.115 | 1.53 [0.90, 2.46] |
| Mother’s age in years (Median, Q1, Q3) | **1.05 [1.01, 1.09]** | **0.014** | 1.05 [1.00 – 1.08] | Non-significant in univariable analysis | | |
| Parental age gap in years: (Median, Q1, Q3) | 0.97 [0.91, 1.03] | 0.365 | 0.97 [0.92 – 1.03] | Non-significant in univariable analysis | | |
| Number of children ever born | 0.96 [0.86, 1.08] | 0.521 | 0.96 [0.87 – 1.07] | **0.84 [0.71, 0.98]** | **0.031** | 0.85 [0.73, 0.98] |
| Birth order | **0.87 [0.01, 0.97]** | **0.013** | 0.88 [0.01 – 0.97] | 0.90 [0.80, 1.02] | 0.091 | 0.91 [0.82, 1.02] |
| Medical complications during pregnancy (gestational hypertension, diabetes, eclampsia and maternal bleeding) | **2.73 [1.31, 5.69]** | **0.008** | 2.23 [1.27, 3.87] | Non-significant in univariable analysis | | |
| Infection during pregnancy (fever, malaria and other infections) | Non-significant in univariable analysis | | | **4.27 [1.20, 15.16]** | **0.025** | 3.21 [1.18, 6.28] |
| Drug misuse during pregnancy | **0.45 [0.20, 0.98]** | **0.045** | 0.48 [0.22, 0.98] | **4.12 [1.08, 15.75]** | **0.039** | 3.14 [1.07, 6.36] |
| Delivery place - home | 0.56 [0.28, 1.10] | 0.092 | 0.58 [0.30, 1.09] | **0.29 [0.13, 0.64]** | **0.002** | 0.31 [0.14, 0.66] |
| Labour and birth complications (induced labour and prolonged labour, PROM, umbilical cord complications and MOH) | 2.83 [0.89, 9.01] | 0.079 | 2.39 [0.90, 5.00] | **6.32 [1.81, 22.03]** | **0.004** | 4.13 [1.67, 7.10] |
| HIE | **9.54 [3.51, 25.97]** | **<0.001** | 5.15 [2.81, 7.43] | 1.00 [1.00, 1.01] | 0.158 | 1.00 [1.00, 1.01] |
| Birth weight in kgs (Mean, SD) | **0.70 [0.49, 0.98]** | **0.040** | 0.72 [0.52, 0.98] | **0.46 [0.23, 0.99]** | **0.047** | 0.49 [0.25, 0.99] |
| Low birth weight (≤ 2.5 kg) | **1.00 [0.99, 1.00]** | **<0.001** | **1.00 [0.99, 1.00]** | 1.00 [0.99, 1.00] | 0.272 | **1.00 [0.99, 1.00]** |
| Seizures at birth | 6.79 [0.84, 54.61] | 0.072 | 4.30 [0.85, 8.59] | 3.35 [0.93, 12.07] | 0.064 | 2.71 [0.94, 5.73] |
| Cerebral malaria anytime in childhood | Not assessed | | | 7.57 [0.95, 60.21] | 0.056 | 4.57 [0.96, 8.70] |
| Neonatal jaundice | Non-significant in univariable analysis | | | **6.56 [1.85, 23.26]** | **0.004** | 4.23 [1.71, 7.21] |
| Head injury | 2.97 [0.77, 11.46] | 0.115 | 2.48 [0.79 – 5.60] | Not assessed | | |
| Seizures before 3 years | **13.00 [3.02, 55.90]** | 0.001 | 5.01 [2.51 – 8.61] | Not assessed | | |

Note: NDD= Neurodevelopmental Disorder, TD= Typically Developing, CI= Confidence Interval, Q1, Q3= Quartile 1, Quartile 3, SD= standard deviation (mean and SD are provided for continuous variables with a normal distribution and median and Q1, Q3 are provided for count variables or continuous variables without normal distribution), SE- standard error, p-values in **bold** <0.05
