# Supplementary material for: Socio-medical factors associated with neurodevelopmental disorders on the Kenyan coast
Source: PLOS Glob Public Health. 2025 May 30;5(5):e0003806. doi: 10.1371/journal.pgph.0003806 (PMC12124531; doi:10.1371/journal.pgph.0003806)
Supplement: Attachment — Submitted filename: ReviewerReport_PGPH-D-24-02176.docx [file pgph.0003806.s002.docx]

Dear Dr. Catherine Kyobutungi and Professor Madhukar Pai,

**RE: Manuscript *Socio-medical Factors Associated with Neurodevelopmental Disorders on the Kenyan Coast***

Thank you for the opportunity to review this manuscript, which reports on a quantitative study exploring natal risk-factors associated with Autism Spectrum Disorder and Intellectual Disability among two samples of children in Kenya. This research appears to be a good fit for the scope of PLOS Global Public Health and addresses an important gap in the literature’s representation of diverse populations in neurodevelopmental disorder research.

I have read the manuscript thoroughly and believe it requires major modifications to meet publishing standards. The main strengths of this manuscript include its use of a relatively large sample of underrepresented participants, comparisons between two independent samples, and robust identification of risk factors. Broadly, the manuscript can be improved by reducing repetition throughout and presenting information in a more structured and specific manner. I provide more detail on these improvements alongside other suggestions below.

**Abstract**

- It is unclear why ADHD is mentioned in the abstract as it is not relevant to the present study, which examines neurodevelopmental disorders as autism and intellectual disability.

**Introduction**

- The introduction does a good job of summarising existing knowledge on the natal risk factors associated with NDDs and identifying gaps in research with individuals from African countries.
- The opening emphasis on prevalence sets the reader up to expect a study on the prevalence of NDDs in African countries.
- The introduction could be strengthened by presenting a summary of what is known about the risk factors broadly, then what is known (and of course not known) for under-represented populations, bringing in the point that socioenvironmental factors that increase the incidence/worsen the impact of the natal risk factors are more prevalent in these regions.
- The final para of the intro should be a high-level description of the current study (i.e., study-specific detail belongs in the methods section) and should include a clear research question.
- Some additional minor points:
  - Line 68, “prevalence of autism has doubled” – over what time period?
  - Line 78, there is an errant period after “toxins”.
  - Line 89/90 “pre-term birth accounts for more than 13% of the global burden of NDDs, and Africa contributes 10% of the burden” – this does not make sense.
  - Line 112 “Using a built-in replication approach” – without further explanation, it’s not clear what is meant by this. I recommend moving this detail to research design in the methods section and expanding.

**Methods**

- The methods section is difficult to follow. In its current presentation, the procedures and materials are unclear. To improve flow and comprehension, I recommend the following structure under a 1^st^-level heading of “Methods”:
  - **Research Design and Setting**
    - I recommend reporting on ethics approvals here (it should be clear immediately that you have ethical approvals).
    - I think it would be helpful to the reader if the methods section opened with a clear explanation of the overarching study design – essentially the information reported on lines 109-112, which then logically flows into description of the settings and recruitment procedures.
    - I recommend 1-3 sentences briefly describing the two studies (as done for the Kilifi study on lines 122-124) and then describing each in more detail under its own heading (ie, one for each of the study samples).
    - From my perspective, the figures visualising the diagnoses in each sample are a distraction and not a good use of space.
  - **Procedures and Measures**
    - By combining procedures and measures, it may be clearer to the reader how diagnoses were determined and reduce repetition. Now, this detail is sort-of reported in a few areas and its unclear at what point and by whom diagnoses were determined.
    - It is not clear whether new data was collected for this present study or if all the data used was pre-collected; it’s also not clear whether the pre-collected data was collected at one time-point or multiple.
    - The consent processes are a little vague. Was assent obtained from child participants?
    - Description of the measures used is insufficient and would be better presented by measure not by study. More detail is needed on the scales used, including their psychometric properties (including current sample where relevant). Perhaps a table would be useful for presenting this information, as it would be possible to clearly illustrate which measures were used where and when.
    - Be specific – “among others” (line 203) is vague and introduces scepticism about what data was collected that isn’t reported on.
    - The section “socio-medical factors of interest” is repetitive. Within the measures section, all variables should be represented. The subsequent grouping of variables should be reported in the data analyses section. I wonder if Table 1 is necessary, given the corresponding data for the samples is reported in Table 2.
  - **Participants**
    - Avoid reporting the same information multiple times. The details reported about participants in the results sections should be removed from this section.
    - A post-hoc power analysis should be conducted to provide insight into what effects would be reasonable to identify given the sample size.
    - I wonder if a justification for the exclusion of individuals with neuro-motor conditions could be included?
    - The authors state, “Given that autism commonly co-occurs with intellectual disability in this setting, we combined the diagnostic groups for analysis”. This phrasing implies that ASD+ID co-occurrence is common generally, but I think the authors mean to say that there is high co-occurrence within their sample. If so, this should be rephrased. If not, it should be cited. Regardless, I’m not convinced by this rationale for collapsing ASD and ID into a single NDD category. Further justification of this decision is needed.
      - Is there robust evidence that the natal pathways to autism and ID are similar and thus modelling risk factors for both is essentially equivalent?
      - Was this decision informed more so by the preferred analysis?

Also, data preparation (like collapsing variables and dealing with missing data) should be reported under the data analysis section.

- **Data Analyses**
  - The first paragraph in this section (lines 235-238) arguably belongs in the measures section. One should not be at the point of data analysis to decide whether the research question is answerable with the data.
  - It is unclear what analyses were conducted for what purpose. I recommend the authors re-write this section to clearly articulate their analytic steps with reasoning.
    - For example, lines 245-248, the authors state “Distribution difference for categorical variables was evaluated using chi-square tests and for analysis of variance for continuous variables compared with analysis of variance” – this makes no sense.
  - The data analysis section should explicitly state what analyses were done for what purpose and with what variables. At present, the authors have not clearly explained their planned analyses. For example, the purpose of the analysis described in lines 249-253 is not given.
  - Perhaps this section could be approached in two parts: 1) data preparation, and 2) data analyses. In the data preparation, cleaning/screening, computing variables, etc. can be described. In data analysis, the steps taken can be described - preferably in a temporally logical order (i.e., descriptives, correlations within sample, group differences between samples, then the logistic regressions).
  - Please provide a citation for FDR strategy.

**Results**

- I think the flow of the descriptive characteristics could be improved by reporting details of each study sample separately under its own 3^rd^-level heading, then reporting on the full sample and the comparisons between samples under a third heading. Now, it’s difficult to follow because information is inconsistently reported.
- Confidence intervals are easier to distinguish by using square brackets rather than parentheses. I’d also consider separating upper and lower bounds with a comma instead of a dash. Reporting of statistics generally needs to be proofed, as there are many inconsistencies in use of punctuation and spacing in statistical sentences.
- Generally, the results section could be made more concise. For example, lines 330-334 is repetitive and wordy.
- Avoid reporting the same information multiple times. Information is presented in-text and in tables. For the benefit of the reader, I recommend deciding which information is best understood in table format and what is best understood in-text and removing repetition. This section of the results could be much more concise. Perhaps a bar graph could be useful here to visualise the sample differences?
- The coefficients for analyses should be reported with p values in Table 2.
- It is not accurate to describe the outcome as “autism” because the category is NDD (e.g., line 339). This needs to be addressed throughout (including in the discussion).
- Stating that a variable is associated with “reduced risk” implies that variable causally reduces risk. It is more accurate to state that the variable is associated with lower risk. Line 339 “most reduced risk” should be “least risk”.
- I don’t think it is appropriate in the results section to characterise an association as “protective (line 341). As the authors rightly discuss, there is no theoretical explanation for why giving birth at home would reduce the risk of NDD (rather, those giving birth at home have fewer natal complications that increase risk).
- Similarly, be mindful of how significance is reported. An association that does not reach the threshold for statistical significance should be described as “non-significant” not “statistically not significant” (line 344).
- I wonder if the rationale for variables controlled through the write-up should be provided?
- Consider reporting the multivariable analysis by variables/construct rather than study – this would make it easier for your reader to understand the results comparatively (which is a strength of this paper).
- Lines 347-349 should be removed as they are repeated on 358-360.
- Line 367, “mother’s age” – is this mothers age at birth or current?
- Line 368, font size changes for confidence intervals.
- Line 369, “and a few others” – be specific in reporting results.

**Discussion**

- The discussion does well to contextualise the current study within the existing literature base. Generally, the authors provide insightful interpretation and critical considerations of the meaning of the results. Leveraging this throughout would further strengthen the discussion. For example, lines 436-439 describe the findings of another study but without interpretation, it’s hard to see the relevance.
- My understanding is that the NeuroDev study is positioned as a “replication study” because it collected data more recently, but I wonder if this is somewhat misleading? Perhaps I have misunderstood, but I interpreted that the NeuroDev study was independent of the Kilifi study but were similar enough to draw group comparisons?
- As mentioned in the results section, it is not accurate to describe the outcome as “autism” because the category is NDD.
- Line 442, it is stated that a result was “successfully replicated in the NeuroDev study” despite it being non-significant in the Kilifi study – therefore I’m not sure it’s accurate to characterise as “replicated” – replicated from where?
- I’m not convinced that maternal education can be considered a risk factor for NDD – this is not a causal association; rather, maternal education is associated with *diagnosis*.
- I appreciate the discussion of foetal involvement in natal processes and experiences. I wonder if this could be further unpacked to clarify a potential interpretation being that autistic children cause difficult births? Additionally, there is scope to provide some suggestions for future research (line 490-491) – more research is always needed, but what is needed to address the specific point being made?
- Line 502 needs a citation.
- The discussion of home delivery and child age are particularly interesting. The authors do well to provide logical explanations, and I would consider stronger language across lines 514-522. I’d argue that the explanations are not just plausible, they are most likely.
- Lines 531-533 are floating uncontextualized.
- It is unclear what is meant by “within-study” approach (line 536). As mentioned above, I may have misinterpreted the link between the NeuroDev and Kilifi studies, but my understanding is that they were independent (i.e., participants were not represented in both). Similarly, I’m not sure what is meant by “reliance on recall” (line 538) – the manner of data collection needs to be clearer in the methods section.
- When reporting limitations, I think it is important to explain what their impact is. What does it mean for our interpretation and subsequent application of the results in light of these limitations? For example, line 540 – what does it mean for this study that there may have been underreporting of certain risk factors, and what risk factors are thought to be underreported?
- I urge the authors to consider what they mean when they say that maternal mental health is an “emerging risk factor”. The terms “mental health” and “psychiatric history” are exceedingly vague compared to the precision of gene markers and highly specific natal risks. I am not suggesting that mental health is unimportant; rather, I am saying that it’s just as important as physical ‘health’ – would it be too vague to say that we need to further investigate “physical health factors”?
- Lines 554 – scope to provide further explanation of why these factors would be important and how they would change our understanding.
- Line 560, again, it’s not a fair representation of the study to focus on the “aetiology of autism”.
- The conclusion could be more impactful by being specific about the most important and consistent risk factors.

**General**

- Throughout the manuscript, the Kilifi Autism Study is referred to in various terms. I recommend introducing it as the Kilifi Autism Study in Methods-Setting, and from there on referring to it as the Kilifi Study consistently. Using different labels/terms for the same thing throughout increases cognitive load.
- I recommend consistently referring to the individuals who participated in this research as “participants” and not ‘cases’.

Warm regards,

Dr Lexy Staniland | Curtin University enAble Institute
