# Supplementary material for: Socio-medical factors associated with neurodevelopmental disorders on the Kenyan coast
Source: PLOS Glob Public Health. 2025 May 30;5(5):e0003806. doi: 10.1371/journal.pgph.0003806 (PMC12124531; doi:10.1371/journal.pgph.0003806)
Supplement: Attachment — Submitted filename: Response to Reviewers_16022025.docx [file pgph.0003806.s004.docx]

**Detailed Response to Reviewers**

**Reviewer #1**

**General comments**

1. The manuscript described the data and conclusions appropriately which was methodologically and ethically acceptable.
2. The statistical analysis was sophisticated and well presented
3. I'm satisfied about the availability of all data.
4. Manuscript was written in clear, grammatically correct standard English.
5. I have no concerns around ethical clearance

**Response:** Thank you very much for taking the time to review our manuscript and for the encouraging and positive feedback shared above. We are glad that the reviewer found the manuscript methodologically sound and well-written.

**Comment 1:** It would be better if the study was done in a younger age group of children. I have noted that the mean age of Kilifi Autism study was 12 years, and the TD group had the mean age was 9 years. I understand that SCQ lifetime was used, but exposure to developmental trauma is a challenge to the diagnosis of ASD in older children.

**Response 1:** Thank you very much for this note. We do hear you and understand the concern about the older age group. In many parts of Africa, the diagnosis of neurodevelopmental disorders occurs around 8-10 years of age (Bakare and Munir, 2011), the average age of 12 for our cohort is consistent with the usual age. We have added the investigation of these risk factors in younger children as a potential avenue for future research. Please see lines 769 to 770 in the tracked changes version, lines 571 to 573 in the clean version, and an excerpt here:

Finally, there is also an opportunity to investigate risk and protective factors in prospective study designs, including younger children, to better understand factors that can connect brain and behaviour change and interventions due to these modifiable factors. ^58^

**Comment 2**: To explore etiology in ASD, I think it's better to assess children only with ASD without intellectual disability in your future studies.

**Response 2:** Thank you for this comment; we agree with you. We have corrected this, as noted by other reviewers as well, that our focus is on the aetiology of neurodevelopmental disorders, including autism and intellectual disability.

**Reviewer #2**

Dear authors,
Thank you for the opportunity to review your manuscript, which addresses an important gap in the literature. I read with interest and hope that my feedback is useful in strengthening the manuscript for publication. Please see my feedback in the attached letter to the editors.

**Response:**

Dear Reviewer,

We greatly appreciate the time, care and thoughtfulness of your detailed response. This substantive piece of feedback has helped in strengthening the manuscript. Please see below detailed responses to your comments.

**Abstract**

1. **Comment:** It is unclear why ADHD is mentioned in the abstract as it is not relevant to the present study, which examines neurodevelopmental disorders as autism and intellectual disability.

**Response:** Thank you very much for this point; we agree this may not be relevant. We have omitted the initial mention of ADHD in the abstract.

**Introduction**

2. **Comment:** The introduction does a good job of summarising existing knowledge on the natal risk factors associated with NDDs and identifying gaps in research with individuals from African countries.

**Response:** Thank you very much for this feedback.

3. **Comment:** The opening emphasis on prevalence sets the reader up to expect a study on the prevalence of NDDs in African countries.

**Response:** Thank you for this feedback. We can see how the discussion on prevalence might prime the reader to expect a study on the prevalence. We have omitted some sentences discussing prevalence. We hope this redirects the focus to the sociomedical risk factors of NDDs. Please see lines 65 to 79 in the tracked version, lines 65 to 72 in the clean version and the excerpt below:

Most NDD studies have been carried out in high-income settings, with a recent review of global autism prevalence only including data from three African countries and other Africa-based studies being smaller-scale case-control studies.^2^ Research indicates that the prevalence of NDDs, such as intellectual disability and autism, in parts of Africa and other lower-middle-income countries (LMICs) may be higher due to the incidence of risk factors for NDDs. These include limited access to healthcare facilities and undernutrition and other risk factors for neuro-behavioural disorders such as adverse perinatal events, infections of the brain and environmental toxins, which are more prevalent in these regions.^3-4^

4. **Comment:** The introduction could be strengthened by presenting a summary of what is known about the risk factors broadly, then what is known (and of course not known) for under-represented populations, bringing in the point that socioenvironmental factors that increase the incidence/worsen the impact of the natal risk factors are more prevalent in these regions.

**Response:** Thank you for this helpful suggestion. We have provided a broad overview of the sociomedical risk factors before the discussion of specific risk factors. Please see lines 83 to 88 in the tracked version and lines 73 to 78 in the clean version. Please also see an excerpt below:

NDDs are a complex group of conditions with environmental and genetic risk factors implicated in its aetiology ^5–7^. Studies show that there are critical periods of neurodevelopment, where exposure to adverse environmental factors, such as viral or bacterial infection of the pregnant mother, exposure to certain medications, and chemical and physical pollutants in the prenatal period, can disrupt typical neurobiological development, and these disruptions may be associated with NDDs ^8–10^.

5. **Comment:** The final part of the intro should be a high-level description of the current study (i.e., study-specific detail belongs in the methods section) and should include a clear research question.

**Response:** Thank you for this point. We have made some changes to the final paragraph as suggested. Please see lines 113 to 119 in the tracked changes copy and lines 102 to 108 in the clean copy. Please also see the excerpt below:

Historically, research on NDDs has focused on the Global North; environmental factors such as infectious diseases during pregnancy and limited healthcare resources during pregnancy would allow the investigation of unique risk and protective factors specific to Africa. These could potentially inform public health strategies towards the early identification of autism in our settings. This study aims to identify prenatal, perinatal and postnatal factors associated with NDDs, such as autism and intellectual disability in Coastal Kenya, comparing children with autism to children with other NDDs as well as typically developing children.

- **Comments:** Some additional minor points:
  - Line 68, “prevalence of autism has doubled” – over what time period?

**Response:** We have incorporated the feedback on the first paragraph being overly focused on prevalence; as such, this sentence has been omitted.

- Line 78, there is an errant period after “toxins”.

**Response:** Apologies, we have deleted this.

- Line 89/90 “pre-term birth accounts for more than 13% of the global burden of NDDs, and Africa contributes 10% of the burden” – this does not make sense.

**Response:** Thank you for pointing this out. We have rephrased this sentence. Please see lines 95 to 97 in the tracked changes version and lines 85 to 87 in the clean version of the manuscript and an excerpt here:

These prenatal factors are more common in LMICs; for example, pre-term births in South Asia and Africa contribute to 80% of the global burden.^11^

- Line 112 “Using a built-in replication approach” – without further explanation, it’s not clear what is meant by this. I recommend moving this detail to research design in the methods section and expanding it.
- **Response:** Thank you, we have deleted this portion from the introduction.

**Methods**

- **Comment:** The methods section is difficult to follow. In its current presentation, the procedures and materials are unclear. To improve flow and comprehension, I recommend the following structure under a 1^st^-level heading of “Methods”:
- **Response:** Thank you for the notes and suggestions in the bullet points below. This is very helpful in the restructuring of the flow of the methods section. We have made all the suggested changes in the points above and below. Please see lines 130 to 391 in the tracked version and lines 109 to 268 in the clean version of the manuscript.
  - **Research Design and Setting**
    - I recommend reporting on ethics approvals here (it should be clear immediately that you have ethical approvals).

**Response:** Thank you for the suggestion; we have moved this section to appear earlier in the methods section as recommended. Please see lines 137 to 143 in the tracked changes version and lines 110 to 129 in the clean version of the manuscript.

- I think it would be helpful to the reader if the methods section opened with a clear explanation of the overarching study design – essentially the information reported on lines 109-112, which then logically flows into a description of the settings and recruitment procedures.

**Response**: Thank you for this note; we have moved the lines to the beginning of the research design and setting sub-heading. Please see lines 132 to 136 in the tracked version of the manuscript and lines 111 to 115 in the clean version of the manuscript.

- I recommend 1-3 sentences briefly describing the two studies (as done for the Kilifi Autism study on lines 122-124) and then describing each in more detail under its own heading (i.e., one for each of the study samples).

**Response**: Thank you for this note; we have made the suggested change. Because of the restructuring of the methods section, and because a lot of the focus is on the procedure, measures and participants, and so that there isn’t repetition, this section includes a high-level description of the two studies with further details in the subsequent sections. Please see lines 131 to 142 in the tracked changes version of the manuscript and lines 110 to 121 in the clean version of the manuscript.

- From my perspective, the figures visualising the diagnoses in each sample are a distraction and not a good use of space.

**Response**: Thank you for this note- we have omitted the graphics.

- **Procedures and Measures**
  - By combining procedures and measures, it may be clearer to the reader how diagnoses were determined and reduce repetition. Now, this detail is sort-of reported in a few areas, and it’s unclear at what point and by whom diagnoses were determined.

**Response**: Thank you for this point; we have made these changes as suggested. Please see lines 202 to 263 in the tracked changes version of the manuscript and lines 122 to 181 in the clean version of the manuscript.

- It is not clear whether new data was collected for this present study or if all the data used was pre-collected; it’s also not clear whether the pre-collected data was collected at one time point or multiple.

**Response**: Thank you for this note. We have clarified this by adding this sentence at the beginning of this section. Please see lines 204 to 205 in the tracked changes version, lines 123 to 124 in the clean manuscript, and here below:

Data collection for the Kilifi Autism study is concluded, while the NeuroDev study collection is ongoing with one-point data collection for both studies.

- The consent processes are a little vague. Was assent obtained from child participants?

**Response**: Thank you for this note; we hear you. We have added a few more details to the consent processes. Please see lines 205-210 in the tracked changes version of the manuscript, lines 124-129 in the clean manuscript and here below:

Information about the study was shared with potential participants in the language the participants were most comfortable in (Kiswahili or Kigiryama). Informed written consent to participate in the study was sought from all parents or caregivers of children with or without NDDs enrolled on the two studies. Verbal assent was requested from all child participants, with written assent requested for all children above 13 years.

- Description of the measures used is insufficient and would be better presented by measure not by study. More detail is needed on the scales used, including their psychometric properties (including current sample where relevant). Perhaps a table would be useful for presenting this information, as it would be possible to clearly illustrate which measures were used where and when.

**Response**: Thank you very much for this note; we have added more information in a tabular format. Please see lines 234 to 232 and lines 262 to 263 in the tracked changes version and lines 151 to 152 and lines 179 to 180 in the clean manuscript. For some of the bespoke questionnaires, such as the sociodemographic and neuromedical questionnaires, we have added a few references from previously done studies that have used these measures.

- Be specific – “among others” (line 203) is vague and introduces scepticism about what data was collected that isn’t reported on.

**Response**: Thank you for this note; as we have added the measures and variables used for analysis in Tables 1 and 2, this comment is now resolved.

- The section “socio-medical factors of interest” is repetitive. Within the measures section, all variables should be represented. The subsequent grouping of variables should be reported in the data analyses section. I wonder if Table 1 is necessary, given the corresponding data for the samples is reported in Table 2.

**Response:** Thank you very much for this comment. We have moved the reporting of the grouping of variables in the data analysis section. Please see lines 312 to 327 in the tracked version and lines 207 to 226 in the clean version of the manuscript. We agree with your comment; with the addition of the tables on the measures, the previous Table 1 is not necessary.

- **Participants**
  - Avoid reporting the same information multiple times. The details reported about participants in the results sections should be removed from this section.

**Response**: Thank you for this comment, this is noted. We have omitted repetitive details about the participants.

- A post-hoc power analysis should be conducted to provide insight into what effects would be reasonable to identify given the sample size.

**Response**: Thank you for this note; we agree this would be a helpful addition. We do think, however, this might best be placed in the data analysis section as we are looking at power to detect effects for analysis used in the study and because the participants’ section comes before the description of the tests used. Please see lines 349 to 354 and 367 to 369 in the tracked version, lines 235 to 240 and lines 252 to 255 in the clean version, and an excerpt of this below:

The Kilifi Autism study, with a sample size of 286 participants, was adequately powered to detect large differences (Cohen’s d = 0.9) in proportions between groups with 100% power for chi-square tests and 99% for the Mann-Whitney U tests. The NeuroDev study, with a sample size of 224 participants, was adequately powered to detect large differences (Cohen’s d = 0.9) in proportions between groups with 100% power for chi-square tests and 96% for the Mann-Whitney U tests.

Both the Kilifi Autism and NeuroDev studies were adequately powered to detect large associations (Cohen’s d = 0.9) between the risk factors and NDDs, with a *post hoc* power calculation of 100% for both studies.

- I wonder if a justification for the exclusion of individuals with neuro-motor conditions could be included.

**Response**: Thank you for this note; we have added the justification for the exclusion of children with primary neuromotor conditions in the NeuroDev study. Please see lines 246 to 251 in the tracked version and lines 163 to 168 in the clean version and in the excerpt below:

Children with NDDs were excluded if they had a co-occurring primary neuro-motor condition such as moderate to severe cerebral palsy. The exclusion of children with a co-occurring primary neuro-motor condition, an indication that the child was not able to walk, was added as some of the assessments such as the Molteno Adapted Scales and the neuro-medical assessment included an examination of gross motor development, which would not be able to be assessed.

- The authors state, “Given that autism commonly co-occurs with intellectual disability in this setting, we combined the diagnostic groups for analysis”. This phrasing implies that ASD+ID co-occurrence is common generally, but I think the authors mean to say that there is high co-occurrence within their sample. If so, this should be rephrased. If not, it should be cited. Regardless, I’m not convinced by this rationale for collapsing ASD and ID into a single NDD category. Further justification of this decision is needed.

**Response**: Thank you for this note. Yes, indeed, what we have noted is the co-occurrence of intellectual disability with autism in the NeuroDev Kilifi cohort. We have rephrased the sentence to reflect this situation. Please see lines 291 to 294 in the tracked version, lines 189 to 191 in the clean version and this excerpt here:

In the NeuroDev Kilifi cohort, we noted that autism co-occurs with intellectual disability, with few participants having a diagnosis of autism alone, as such, we combined the diagnostic groups for analysis into children with an NDD vs typically developing children.

- Is there robust evidence that the natal pathways to autism and ID are similar and thus modelling risk factors for both is essentially equivalent?

**Response**: Thank you very much for this note. Autism and intellectual disability are conditions characterised by differences in neurodevelopment, and while these conditions are heterogeneous, their grouping as conditions on the neurodevelopmental continuum has a biological basis as well. These two conditions are comorbid, with about 30-40% of autistic individuals also having an intellectual disability, this co-occurrence may indicate a shared biological pathway (Shenouda et al., 2023). Genetic and environmental risk factors have implicated specific biological pathways in NDDs, these include metabolic disruptions, altered neurodevelopment and interference in cell proliferation and/or migration, which then lead to brain abnormalities that result in cognitive and behavioural disabilities such as NDDs. Genetic variations in genes about 117 genes, including NRXN1, CNTNAP2, NLGN4, SHANK2 and SHANK1, have been definitively linked to both autism and ID (Rooney Riggs, 2022). Studies have also found that perinatal events such as preterm birth and low birth weight are associated with autism + ID (Singh et al., 2013; Schieve et al., 2015). Thus, we think this is sufficient biological and epidemiological evidence is present to justify the studying of these two conditions. We have added a sentence to justify this grouping of autism and ID as our outcome of interest (NDDs). Please also see lines 300 to 306 in the tracked changes version and lines 198 to 204 for an expansion of this. Please also see the excerpt below:

Prenatal factors such as severe maternal obesity are associated with autism (with or without ID). ^37^ A study by Schieve and colleagues found that perinatal factors such as preterm birth, low birth weight and low APGAR scores were associated with autism, ID and autism with co-occurring ID. ^38^ There is plausible biological and epidemiological evidence that supports the grouping of autism and intellectual disability as conditions on the neurodevelopmental continuum as discussed in the Research Domain Criteria and adds credence to the investigation of risk factors of these two conditions grouped as NDDs. ^39^

- Was this decision informed more so by the preferred analysis?

**Response**: Yes, this was also another reason we grouped the participants together. We do not have as many children with autism alone in our cohort (due to some of the factors discussed or alluded to above, such as who is easily identified), which was also a reason for the combining of the cohort into NDD vs Typically Developing. We have noted this as well in the participants section, lines 291 to 300 in the tracked version, lines 189 to 198 in the clean version of the manuscript and an excerpt here:

In the NeuroDev Kilifi cohort, we noted that autism co-occurs with intellectual disability, with few participants having a diagnosis of autism alone, as such, we combined the diagnostic groups for analysis into children with an NDD vs typically developing children. While there are strong health and education resources available to tease apart global developmental delays and intellectual disability, particularly those requiring moderate or high support needs, there are still a lot of resources needed to strengthen these systems in the identification of autistic individuals with low or moderate support needs ^34^. This finding is similar to others on the African continent, such as clinical settings in Nigeria, where more severe presentations of autism are seen with an overrepresentation of non-verbal cases as well as co-occurring intellectual disability and ADHD ^35,36^

- Also, data preparation (like collapsing variables and dealing with missing data) should be reported under the data analysis section.

**Response**: Thank you for this note. We have moved the data preparation to the data analysis section. Please see lines 308 to 332 in the tracked version and lines 206 to 226 in the clean version.

- **Data Analyses**
  - The first paragraph in this section (lines 235-238) arguably belongs in the measures section. One should not be at the point of data analysis to decide whether the research question is answerable with the data.

**Response**: Thank you for this point. We have moved this section to the procedures and measures section. Please see lines 212 to 214 in the tracked version and lines 130 to 132 in the clean version.

- It is unclear what analyses were conducted for what purpose. I recommend the authors re-write this section to clearly articulate their analytic steps with reasoning.
  - For example, lines 245-248, the authors state “Distribution difference for categorical variables was evaluated using chi-square tests and for analysis of variance for continuous variables compared with analysis of variance” – this makes no sense.

**Response**: Thank you for this point. We have rewritten the data analysis section and made clear what analysis we carried out with reasoning as to the analysis. Please see lines 333 to 382 in the tracked version and lines 227 to 268 in the clean version.

- The data analysis section should explicitly state what analyses were done for what purpose and with what variables. At present, the authors have not clearly explained their planned analyses. For example, the purpose of the analysis described in lines 249-253 is not given.

**Response**: Thank you for this note. We have added these details to the data analysis section as suggested in the comment above as well; we hope the section is clearer. Please see lines 333 to 382 in the tracked changes version and lines 227 to 268 in the clean version, for the rewritten data analysis section.

- Perhaps this section could be approached in two parts: 1) data preparation, and 2) data analyses. In the data preparation, cleaning/screening, computing variables, etc. can be described. In data analysis, the steps taken can be described - preferably in a temporally logical order (i.e., descriptives, correlations within sample, group differences between samples, then the logistic regressions).

**Response**: Thank you, we have adopted the suggested approach in the rewriting of the data analysis section. Please see lines 307 to 382 in the tracked version, lines 205 to 268 in the clean version of the manuscript.

- Please provide a citation for FDR strategy.

**Response:** Our apologies for the omission. We have added the citation, please see line 372 in the tracked version and line 258 in the clean version.

**Results**

- I think the flow of the descriptive characteristics could be improved by reporting details of each study sample separately under its own 3^rd^-level heading, then reporting on the full sample and the comparisons between samples under a third heading. Now, it’s difficult to follow because information is inconsistently reported.

**Response**: Thank you for this note. We have reworked the descriptive characteristics section with the suggested structure of reporting by study. Please see lines 399 to 441 in the tracked version and lines 271 to 301 in the clean version.

- Confidence intervals are easier to distinguish by using square brackets rather than parentheses. I’d also consider separating upper and lower bounds with a comma instead of a dash. Reporting of statistics generally needs to be proofed, as there are many inconsistencies in use of punctuation and spacing in statistical sentences.

**Response**: Thank you for this note; our apologies for this. We have proofed the results section, corrected the inconsistencies, and used square brackets, separating upper and lower bounds with a comma.

- Generally, the results section could be made more concise. For example, lines 330-334 is repetitive and wordy.

**Response**: Thank you for this note; we have removed lines 330-334 and made efforts to make the results section more concise.

- Avoid reporting the same information multiple times. Information is presented in-text and in tables. For the benefit of the reader, I recommend deciding which information is best understood in table format and what is best understood in-text and removing repetition. This section of the results could be much more concise. Perhaps a bar graph could be useful here to visualise the sample differences?

**Response:** Thank you for this note. We have gone over the results section and removed the table with the multivariable analyses, presenting this as a supplementary table and the main results for this analysis as text.

- The coefficients for analyses should be reported with p values in Table 2.

**Response**: Thank you for this note. For the descriptives table, we used chi-square tests and Mann-Whitney U tests to primarily assess associations or differences between the NDD and typically developing group as such we do not have coefficients to present. We have added a legend that was missing describing the tests we used to assess the differences between groups, I hope this is helpful. Please see lines 444 to 446 in the tracked changes and lines 304 to 306 in the clean version.

- It is not accurate to describe the outcome as “autism” because the category is NDD (e.g., line 339). This needs to be addressed throughout (including in the discussion).

**Response**: Our apologies for the error. This is an oversight on my part as we were to change the outcome to NDDs. This has been done consistently in the manuscript.

- Stating that a variable is associated with “reduced risk” implies that the variable causally reduces risk. It is more accurate to state that the variable is associated with lower risk. Line 339 “most reduced risk” should be “least risk”.

**Response**: Thank you for this note. We have changed this to “least risk” as suggested. Please see line 460 in the tracked version and line 314 in the clean version. We have also consistently changed this across the manuscript.

- I don’t think it is appropriate in the results section to characterise an association as “protective (line 341). As the authors rightly discuss, there is no theoretical explanation for why giving birth at home would reduce the risk of NDD (rather, those giving birth at home have fewer natal complications that increase risk).

**Response**: Thank you for this note; we have removed all references to protective factors.

- Similarly, be mindful of how significance is reported. An association that does not reach the threshold for statistical significance should be described as “non-significant” not “statistically not significant” (line 344).

**Response**: Thank you for this note, we have corrected the reporting of non-significant results.

- I wonder if the rationale for variables controlled through the write-up should be provided?

**Response**: Thank you for this note. We have added a justification of the sociodemographic variables such as parental education and ethnicity as control variables in the data analysis section. Please see lines 364 to 367 in the tracked version and lines 249 to 252 in the tracked version, as well as the excerpt below:

Sociodemographic factors such as parental education have been strongly linked to child health and development outcomes.^41,42^ Studies in the United States, India and China have found that higher parental education is associated with an earlier autism diagnosis.^43–45^

- Consider reporting the multivariable analysis by variables/construct rather than study – this would make it easier for your reader to understand the results comparatively (which is a strength of this paper).

**Response**: Thank you for this note. We have restructured the multivariable analysis by variable and omitted the table in the main text as this would be a repetition of what is written in the results section; we have, however, added it as a supplementary table. Please see lines 479 to 521 in the tracked version of the manuscript and lines 330 to 371 in the clean version of the manuscript.

- Lines 347-349 should be removed as they are repeated on 358-360.

**Response**: Thank you for this note; we have removed these lines.

- Line 367, “mother’s age” – is this mothers age at birth or current?

**Response**: Thank you for this note; we have clarified that this is both ages. Please line 426-427 in the tracked changes version of the manuscript and lines 291-292 in the clean version:

The difference in the ages, both currently and at birth, is not statistically different (p=0.560).

- Line 368, font size changes for confidence intervals.

**Response**: Thank you for this note, all font size in the manuscript is consistent.

- Line 369, “and a few others” – be specific in reporting results.

**Response**: Thank you for this note; we have reported all the significant and non-significant results.

**Discussion**

- The discussion does well to contextualise the current study within the existing literature base. Generally, the authors provide insightful interpretation and critical considerations of the meaning of the results. Leveraging this throughout would further strengthen the discussion. For example, lines 436-439 describe the findings of another study but without interpretation, it’s hard to see the relevance.

**Response**: Thank you for this feedback. We have added more interpretation of some of the studies mentioned in lines 436-439 of the older version of the manuscript. Please see lines 600 to 607 in the tracked version and lines 427 to 434 in the clean version of the manuscript. Please see an excerpt here below as well:

There is concern that synthetic oxytocin administered during labour may enter the foetal circulation system, deactivate oxytocin receptors and disrupt the oxytocinergic system, thus increasing the risk for NDDs such as autism and ADHD. ^56,57^ While both positive and null findings of this association have been reported, a systematic review in 2018 indicates that the link between synthetic oxytocin and NDDs is weak, with a call for further studies investigating oxytocin-stimulated labour and NDDs to account for maternal psychopathology and including sibling comparison in the study design.

- My understanding is that the NeuroDev study is positioned as a “replication study” because it collected data more recently, but I wonder if this is somewhat misleading? Perhaps I have misunderstood, but I interpreted that the NeuroDev study was independent of the Kilifi study but were similar enough to draw group comparisons?

**Response**: Thank you for this note. Yes, indeed, your understanding is correct, NeuroDev is a more recent study. We have attempted to make this clearer when describing the data analysis approaches as seen in lines 139 to 142 and lines 111 to 114. We hope this will make it clearer in the discussion section.

- As mentioned in the results section, it is not accurate to describe the outcome as “autism” because the category is NDD.

**Response**: Thank you for this note, we have corrected this throughout the manuscript.

- Line 442, it is stated that a result was “successfully replicated in the NeuroDev study” despite it being non-significant in the Kilifi study – therefore I’m not sure it’s accurate to characterise as “replicated” – replicated from where?

**Response**: Thank you for this note; we agree and have corrected this sentence. Please see line 571 in the tracked changes version and line 398 in the clean version.

- I’m not convinced that maternal education can be considered a risk factor for NDD – this is not a causal association; rather, maternal education is associated with *diagnosis*.

**Response**: Thank you for this note; we agree with you. We discussed maternal education in the context of it as a factor adjusted for in the multivariable analysis and as a variable associated with the risk factors analysed. We have made this clear in the discussion section by moving it to the bottom of the risk factors section and also emphasised that maternal education is associated with an NDD diagnosis, not the group of the conditions itself. Please see lines 683 to 691 in the tracked changes version and lines 500 to 508 in the clean version and in an excerpt here:

Maternal education, one of the parental factors that was adjusted for in the multivariable analysis, is important to investigate fully. Studies have found that parental educational level and other sociodemographic markers may affect the awareness of NDDs and help-seeking behaviour due to concerns about their child’s development.^79^ In our analysis, maternal education was associated with delivery place; we see that mothers with higher education levels gave birth in hospitals as opposed to giving birth at home. We also see an association between maternal education level and NDD diagnosis, with mothers of children with an NDD diagnosis having higher education levels, supporting the link between maternal education and developmental concerns.

- I appreciate the discussion of foetal involvement in natal processes and experiences. I wonder if this could be further unpacked to clarify a potential interpretation being that autistic children cause difficult births? Additionally, there is scope to provide some suggestions for future research (line 490-491) – more research is always needed, but what is needed to address the specific point being made?

**Response**: Thank you for this note, we understand the concern regarding the potential interpretation that autistic children cause difficult births. We have added more context and unpacked this statement a little more, explaining that shared genetic and familial factors are what may be implicated in labour complications and autism. Please see lines 655 to 671 in the tracked changes and lines 473 to 488 in the clean version, and this excerpt below:

Children with NDDs often will have mothers who have experienced more pregnancy and birth complications compared to their unaffected siblings and typically developing children. Recent research has also focused on the foetus' involvement in the birth process, with studies finding that the foetus actively engages in the birth process through various physiological mechanisms.^72^ Various hypotheses attempt to explain this observation, two of these competing hypotheses include that NDDs such as autism are aetiologically heterogeneous due to genetic and environmental factors such as birth and labour complications, the second hypothesis is that genetic and familial factors increase the risk of both autism and birth and labour complications, as evidenced by findings in conditions such as Prader-Willi syndrome and Down syndrome. ^73,74^ Researchers also discuss a third hypothesis: in the presence of gene-environment interactions, birth and labour complications may play a role in autism and NDDs, with the modulation of environmental risk factors due to familial liability as seen in meta-analysis of twin studies.^75^ These studies highlight the entanglement between genetic, familial and environmental factors, with more studies needed using various family structures such as twin studies, and studies of non-affected family members to investigate this interplay.

- Line 502 needs a citation.

**Response**: Thank you for this note; we have added a citation. Please see line 682 in the tracked version and line 499 in the clean version.

- The discussion of home delivery and child age is particularly interesting. The authors do well to provide logical explanations, and I would consider stronger language across lines 514-522. I’d argue that the explanations are not just plausible, they are most likely.

**Response**: Thank you for this note. We erred on the side of caution with these explanations, we have worded them stronger now. Please see lines 702 to 720 in the tracked version and lines 517 to 531 in the clean version.

- Lines 531-533 are floating uncontextualized.

**Response**: Thank you for this note, we have moved these lines to the strengths and limitations section of the manuscript, they are more apt in this section following the limitations of the study. Please see lines 769 to 771 in the tracked changes version and lines 569 to 571 in the clean version.

- It is unclear what is meant by “within-study” approach (line 536). As mentioned above, I may have misinterpreted the link between the NeuroDev and Kilifi studies, but my understanding is that they were independent (i.e., participants were not represented in both). Similarly, I’m not sure what is meant by “reliance on recall” (line 538) – the manner of data collection needs to be clearer in the methods section.

**Response**: Thank you for this observation. We have removed reference to the “within-study” approach and added that we used two independent datasets. Please see line 733-734 in the tracked version and lines 533-534 in the clean version.

Thank you for the note on recall data. Here, we meant the reliance on parental report data, which is subject to recall bias. We have made this clear in lines 731 to 732 in the tracked changes version and lines 535 to 537 in the clean version, and here below:

There are some limitations, particularly with the reliance on parental reports, which might lead to some recall bias as opposed to precise measurement data on health cards and medical reports.

- When reporting limitations, I think it is important to explain what their impact is. What does it mean for our interpretation and subsequent application of the results in light of these limitations? For example, line 540 – what does it mean for this study that there may have been underreporting of certain risk factors, and what risk factors are thought to be underreported?

**Response**: Thank you very much for this note, we agree it is important to add the impact of the limitations. We had explained these issues for some limitations but not all, we have added these; please see lines 735-755 in the tracked version and lines 536 to 555 in the clean version and excerpt here:

This may have led to over or under-reporting of certain risk factors such as birth asphyxia and HIE. This may lead to the true link between these factors and NDDs being underestimated or overestimated, leading to biased associations.

This may have led to some slight discrepancies in comparison between cases from both studies, however, to mitigate this we combined the specific NDD diagnoses to a more agreeable definition of case status as a diagnosis of an NDD.

Because of this, the subtleties in the association between these specific risk factors and the outcome may have gone undetected or there may have been a bias in the estimates reported.

- I urge the authors to consider what they mean when they say that maternal mental health is an “emerging risk factor”. The terms “mental health” and “psychiatric history” are exceedingly vague compared to the precision of gene markers and highly specific natal risks. I am not suggesting that mental health is unimportant; rather, I am saying that it’s just as important as physical ‘health’ – would it be too vague to say that we need to further investigate “physical health factors’?

**Response**: Thank you very much for this note; we see your point. We have added more specific recommendations in terms of mental health, psychiatric history and its links to NDDs. Please see lines 756 to 760, lines 556 to 560 in the clean version and an excerpt here:

It would have been useful to investigate risk factors such as maternal mental health and general parental psychiatric history, which is a significant confounding factor for perinatal risk factors and NDDs. Studies have found that both maternal and parental prenatal mood and anxiety disorders, maternal eating disorders, and exposure to prenatal stress are significantly linked to autism and ADHD. ^80–82^

- Lines 554 – scope to provide further explanation of why these factors would be important and how they would change our understanding.

**Response**: Thank you for this note; we have added explanations as to why the stated factors would be important to study. Please see lines 760 to 771 in the tracked version, lines 560 to 571 in the clean version and an excerpt here:

We were also unable to incorporate genetic results and other potential biomarkers, such as neuroimaging. The addition of genetic approaches would provide deeper insights into the biological mechanisms of NDDs and potentially evaluate gene-environment interactions while the incorporation of neuroimaging would reveal structural, functional or connectivity differences in the brain associated with NDDs. We were also missing information on other biological measures of infection and inflammation in pregnancy and nutritional factors, such as iron deficiency in pregnancy and early childhood. These factors would also help identify pathways linking or disruptions of critical pathways, for example, with maternal immune activation, to neurodevelopmental outcomes. Finally, there is also an opportunity to investigate risk and protective factors in prospective study designs, including younger children, to better understand factors that can connect brain and behaviour change and interventions due to these modifiable factors. ^58^

- Line 560, again, it’s not a fair representation of the study to focus on the “aetiology of autism”.

**Response**: Thank you for this note, our apologies, we have made the change to the aetiology of NDDs.

- The conclusion could be more impactful by being specific about the most important and consistent risk factors.

**Response**: We have added the important and consistent risk factors in the conclusion section. Please see lines 773 to 775 in the tracked version and lines 573 to 575 in the clean version and this excerpt here:

In conclusion, the study identified labour and birth complications, HIE, neonatal jaundice and seizures before age 3 as pervasive significant risk factors associated with NDDs. These factors were significant in either the NeuroDev or Kilifi Autism studies, not both.

**General**

- Throughout the manuscript, the Kilifi Autism Study is referred to in various terms. I recommend introducing it as the Kilifi Autism Study in Methods-Setting, and from there on referring to it as the Kilifi Study consistently. Using different labels/terms for the same thing throughout increases cognitive load.

**Response:** Thank you very much for this note; we would like to ask that we leave this as the Kilifi Autism study and use it consistently across the manuscript. This is because the NeuroDev study was also carried out in Kilifi County. We have kept this wording consistent throughout the manuscript. Please let us know if this is acceptable.

- I recommend consistently referring to the individuals who participated in this research as “participants” and not ‘cases’.

**Response**: Thank you for this note. We have made this change as suggested.

**Reviewer #3:**

**Comment:** Summary of the research and overall impression

The manuscript presents the results of original research into the socio-medical factors associated with neurodevelopmental disorders in Kenya. This is a neglected area of public health, particularly on the African continent. The authors demonstrated a good grasp of the relevant literature, which was used to set the scene in relation to the burden of neurodevelopmental disorders. They outlined the peculiarity of the African socio-medical context, justifying the need to generate valid local epidemiological data to inform policymaking, practice and research.
The methods were adequately described, and the conclusions are appropriately supported by the data presented.
Overall, the authors engaged well with the topic in the conduct of the research and reporting of the study results and meet criteria for publication in PLOS Global Public Health.

**Response:** We want to express our gratitude to the reviewer for their thoughtful and encouraging comments on our manuscript. We are pleased that the reviewer found the manuscript engaged well with the topic of neurodevelopmental disorders.

2. Discussion of specific areas for improvement

While the manuscript is highly commendable for the above reasons, I have highlighted areas for improvement by addressing some minor issues:
1. Introduction: The introduction section is very thorough, the authors demonstrating a great understanding of the global and local landscape of neurodevelopmental conditions. However, I have made the following observations:
**Comment 1:** The point made in Line 78, “The same may be true for the prevalence of autism in Africa which is thought to be similar to the global estimate or possibly greater given the high incidence of neuro-behavioural disorders such as adverse perinatal events, infections of the brain and environmental toxins” appears to be a repetition of the point already made in Line 74. Perhaps this should be deleted.

**Response 1**: Thank you for this point. We agree and have deleted this sentence.

**Comment 2:** For the statement made in Line 91, “This has changed little in the last decade”, it will be helpful to provide a reference if available.

**Response 2**: Thank you for this point; we have added a reference. Please see line 98 in the tracked changes version and line 87 in the clean version.

2. Results:
- **Comment 3**: In Line 293, … “and for the NeuroDev study, 151 children with an NDD and 73 typically developing controls with a median age of 11 years (9,13)”, typically developing children included as controls was n=73 but it is n=71 in Table 2. The authors need to correct this.

**Response 3**: Thank you very much for this point. Our apologies for the mistake. We have corrected the Ns in the text and the tables.

**Comment 4**: In Line 296, the authors state, “In the Kilifi Autism study, 41.8% of mothers did not have any formal schooling and 42.4% in the NeuroDev study, in comparison to 13.2% of fathers and 14.8% of fathers in the two studies, respectively” The proportions reported on Table 2 are different: 41.8% of mothers did not have any formal schooling in the Kilifi Autism Study and 42.4% in the NeuroDev study. The authors need to check and reconcile this.

**Response 4**: Thank you very much for this note and our apologies for this error. We have reconciled the proportions mentioned in the tables and the text, which are a little different to what you’ve written here as well. Please see lines 410 to 411, lines 427 to 428 in the tracked changes version of the manuscript and lines 278 to 279 and 292 to 293 in the clean version. Please also see an excerpt below:

Kilifi Autism Study - 36.4% of mothers and 10.1% of fathers did not have formal schooling.

NeuroDev study - 14.3% of fathers and 42.4% of mothers did not have formal schooling.

**Comment 5:** In Line 298, similarly, the proportions of fathers without any formal schooling in the Kilifi Autism Study and the NeuroDev studies are 3.2% of fathers and 14.8% of fathers in the two studies, respectively. These proportions are different in Table 2: These are 9.3% and 14.0% of fathers in the Kilifi Autism Study and the NeuroDev study respectively. The authors should also check and reconcile these.

**Response 5**: Thank you very much for this note, and our apologies for this error. We have reconciled the proportions mentioned in the tables and the text. Please see lines 410 to 411, lines 427 to 428 in the tracked changes version of the manuscript and lines 278 to 279 and 292 to 293 in the clean version and in the clean version and the excerpt above.

**Comment 6:** In Line 306, the statement, “For the NeuroDev study, we see that the ages of the NDD group mothers are 26 years old, and mothers of controls have a median age of 29 years” was already made earlier in Line 303. The authors should delete the second statement.

**Response 6**: Thank you very much for this note. We have omitted the repeated statement.

3. Discussion
**Comment 7:** In Line 403, the statement, “Maternal age (older) in the Kilifi Autism study and labour and birth complications, infection during pregnancy, neonatal jaundice, cerebral malaria anytime in childhood and previous hospitalisation in childhood emerged as statistically significant risk factors associated with autism in the NeuroDev study” based on the results presented, should read, “Maternal age (older) in the Kilifi Autism study and labour and birth complications, infection during pregnancy, neonatal jaundice, cerebral malaria anytime in childhood and previous hospitalisation in childhood in the NeuroDev study, emerged as statistically significant risk factors associated with autism.

**Response 7**: Thank you for this suggested change; we have amended the sentence as suggested. Please see lines 564 to 568 in the tracked changes version and lines 391 to 394 in the clean version.

**Comment 8:** Line 492: the space should be deleted.

**Response 8:** Thank you, we have deleted the extra space.

**Comment 9:** Statements on Line 511, “The protective effect observed with delivery at home in our study warrants more disentangling, as it may suggest that mothers of children with NDDs tend to have higher education levels and may be aware of the potential of obstetric complications during birth, leading them to give birth in a hospital setting” is essentially the same as the statement in Line 514. The authors should reconcile this.

**Response 9:** Thank you very much for this note. We have omitted the repeated statement.

**Comment 10:** In Line 524, “The association of earlier order of birth order and reduced risk for NDD is consistent with findings from another study..” should read, “ The association of earlier birth order and reduced risk for NDD is consistent with findings from another study..

**Response 10:** Thank you for this note. We have rephrased the statement as suggested. Please see lines 697 in the tracked version and line 512 in the clean version.
